# Mental health and its influencing factors among left-behind children in South China: a cross-sectional study

**DOI:** 10.1186/s12889-019-8066-5

**Published:** 2019-12-23

**Authors:** Xiu Zhang, Mengjie Li, Li Guo, Yanna Zhu

**Affiliations:** 1 Department of Maternal and Child Health, School of Public Health, Sun Yat-sen University, No.74 Zhongshan Road II, Guangdong Province, Guangzhou, 510080 China; 2 Present address: Guangzhou Brain Hospital, Guangzhou, Guangdong China; 3 Department of Maternal and Child Health and Sun Yat-sen Global Health Institute, School of Public Health and Institute of State Governance, Sun Yat-sen University, Guangzhou, 510080 Guangdong China

**Keywords:** Left-behind children, Mental health, Community, Place of residence, Age

## Abstract

**Background:**

With rapid development of China’s economy, there were over 68.7 million left-behind children (LBC) in China whose mental health has become a problem of public concern. The present cross-sectional study aimed to investigate the status of mental health and its associated factors of LBC aged 3–16 years old in both rural and urban areas.

**Methods:**

A total of 4187 children (aged 3–16), including 1471 LBC and 2716 non-left-behind children (NLBC), were recruited from 50 communities (22 in urban areas and 28 in rural areas) in Guangdong, China in August, 2014. The mental health problems were assessed using the Strength and Difficulties Questionnaire (SDQ).

**Results:**

No statistically significant difference of SDQ subscales scores about difficulties were found between LBC and NLBC on the whole participants as well as in rural areas or in urban areas within the same age group after adjustments were made (all *p* > 0.05). However, compared with NLBC in the same areas, urban LBC tended to have higher prosocial behaviours scores, while rural LBC had the lowest prosocial behaviours scores not only in the whole age group but also in different age subgroups (*p* < 0.05). Besides, compared with urban LBC, rural LBC were not worse in SDQ subscales scores except for prosocial behaviour at 7–9 age group (*p* = 0.003). Furthermore, higher paternal educational level and longer duration of parental absence, were associated with less difficulties in both rural and urban LBC. Besides, shorter duration of talk per-time but higher communication frequency were associated with less difficulties in rural LBC.

**Conclusions:**

The present study demonstrated that in general, no difference of mental health problems were found between LBC and NLBC. Besides, longer duration of parental absence, shorter duration of talk per time but more communication frequency, and higher paternal educational level tend to have better development of mental health. The findings reinforce the importance of the stability of caregivers and the effective parent-child communication for Chinese rural LBC.

## Background

Since the reform and opening-up policy in late 1970s in China, the rapid economic growth has resulted in the urban-rural income inequality in China. A large number of surplus rural labour swarmed into cities seeking for better employment opportunities, leaving their children at home with a single parent, or relatives [1]. Meanwhile, with the increasing frequency of the talent flow, the number of urban children with one or two migrant parent has been increasing dramatically in recent decades [1]. Those who were younger than 17 years old, left behind at home by one or two migrant parents for at least 6 consecutive months were called Left-behind children (LBC) [2]. In 2015, there were over 68.7 million LBC in China, of which over 54.9 million lived in rural areas, and nearly three quarters aged under eleven. The number of LBC living in urban areas has increased dramatically — nearly 5.7 folds since 2000, reaching 13.8 million [1].

The impact of being left-behind on the development of children is complex. On one hand, parental migration usually means an increasing of family income. On the other hand, it also means the change of main caregivers and a weakened connection between family members. A study in Anhui province showed no significant difference on mental health problems between rural preschool-aged LBC with two migrant parents and non-left-behind children (NLBC) [3]. But numerous studies have found higher prevalence of psychological [4] and behavioural problems [5, 6] in LBC than in NLBC in all age groups [4, 7, 8]. For example, LBC at older age were more likely to suffer from negative emotional experiences like depression [9], anxiety [7], loneliness [10] and neglect [11, 12], and have more conduct problems, including smoking [13] and alcohol consumption [14]. Besides, increasing number of left-behind adolescents were also reportedly sensitive, hostile and paranoid in their interpersonal relationship [15]. Rural LBC in pre-school were found to have lower level of socialization development than that of rural NLBC and children lived in urban areas [16]. However, most existing studies only focused on a single age group, and they failed to cover the whole age range including pre-schooler (under the age of six) and school-aged LBC (aged 7–17).

Additionally, series of studies have demonstrated that child age, sex, family monthly income, age of separation, duration of parental absence, migration type (father only, mother only, both parents) and number of siblings, etc., were associated with LBC’s emotional and behavioural development [17, 18]. For example, Liu Z, etc. [19] revealed that children, separated from parents at a younger age, were more likely to be anxious or depressive, especially those left-behind by either mother or both parents. Besides, urban LBC were reported to have higher rate of internet-addiction than urban NLBC [20]. However, few studies covered rural and urban LBC, and it is still unknown whether their influencing factors are different or not.

Taking all the above into consideration, the present study aimed at investigating the status of mental health and its influencing factors among LBC aged 3 to 16 years by comparing LBC with NLBC within a large sample, and examining whether place of residence (rural and urban) would influence LBC’s mental health development differentially.

## Method

### Participants

With the help of Guangdong Women’s Federation (a government administration in China) in August 2014, participants were enrolled from 50 communities in Guangdong, a province with the largest number of LBC in South China [1]. Inclusion criteria for the participants included 1) aged 3–16 years; 2) with the experience of separate from one or two migrant parents for at least six consecutive months before August 2014; 3) living in the place where the family residence was registered. Inclusion criteria for the control group included 1) aged 3–16 years; 2) living with both parents in the place where the family residence was registered. Exclusion criteria for all participants contained: 1) with a history of serious neurological systemic or mental disease; 2) with obvious physical defects. Questionnaires were obtained from 4334 children, among which 127 questionnaires were invalid and therefore eliminated. Finally, the sample size fell to 4187, including 1471 LBC (765 boys and 687 girls) and 2716 NLBC (1463 boys and 1230 girls) (Table 1).

**Table 1:** Baseline characteristics of LBC and NLBC

Variables	LBC	NLBC	all	*P*
	(*n* = 1471)	(*n* = 2716)	(n = 4187)	
Sex, n(%)				
boys	765(52.7)	1463 (54.3)	2228 (53.2)	0.312
girls	687 (47.3)	1230 (45.7)	1917 (45.8)	
Age (years) mean (SD) ^a^				
3–16 years	8.46 (3.50)	8.52 (3.66)	8.50 (3.60)	0.611
3–6 years	5.04 (1.12)	4.87 (1.17)	4.93 (1.16)	
7–9 years	8.41 (0.84)	8.36 (0.82)	8.38 (0.82)	
10–16 years	12.70 (1.88)	12.78 (1.86)	12.75 (1.87)	
Place of residence, n(%)				
ural	1276 (86.7)	1503 (55.3)	2779 (66.4)	< 0.001
urban	195 (13.3)	1213 (44.7)	1408 (33.6)	
Only child, n(%)				
yes	309 (21.0)	1274 (46.9)	1583 (37.8)	< 0.001
no	1162 (79.0)	1442 (53.1)	2604 (62.2)	
Father’s educational level, n(%)				
primary school or below	185 (12.7)	111 (4.2)	296 (7.1)	< 0.001
middle school	833 (57.4)	908 (34.1)	1741 (41.6)	
high school	303 (20.9)	889 (33.4)	1192 (28.5)	
junior college or bachelor	125 (8.6)	707 (26.5)	832 (19.9)	
master or higher	5 (0.3)	49 (1.8)	54 (1.3)	
Mother’s educational level, n(%)				
primary school or below	210 (14.8)	193 (7.2)	403 (9.6)	< 0.001
middle school	876 (61.7)	966 (36.2)	1842 (44.0)	
high school	237 (16.7)	847 (31.7)	1084 (25.9)	
junior college or bachelor	94 (6.6)	635 (23.8)	729 (17.4)	
master or higher	3 (0.2)	27 (1.0)	30 (0.7)	
Marital status, n(%)				
spinsterhood	9 (0.6)	17 (0.6)	26 (0.6)	0.081
married	1369 (95.1)	2581 (96.6)	3950 (94.3)	
divorced	29 (2.0)	40 (1.5)	69 (1.6)	
widowed	17 (1.2)	16 (0.6)	33 (0.8)	
remarried	16 (1.1)	17 (0.6)	33 (0.8)	
Monthly income (RMB), n(%)				
< 2000	375 (26.2)	307 (11.5)	682 (16.3)	< 0.001
2000–5000	698 (48.8)	1131 (42.4)	1829 (43.7)	
5001–8000	160 (11.2)	696 (26.1)	856 (20.4)	
8001–12,000	45 (3.1)	219 (8.2)	264 (6.3)	
> 12,000	17 (1.2)	102 (3.8)	119 (2.8)	
unknown	134 (9.4)	215 (8.1)	349 (8.3)	

*Note. LBC* left-behind children *NLBC* non-left-behind children

*P* < 0.05 *LBC* vs. *NLBC* assessed by chi-square test for categorical variables unless otherwise indicated. ^a^ Assessed by the unpaired Student t-test

### Sample size calculation

The aim of the present study is to explore the status of mental health among LBC aged 3 to 16 years in China. A α error of 0.05 and a power of 90% (two-sided test) were adopted in statistical analysis. For linear regression, the minimum number of cases included in the study was calculated by the formula N = [(t_α/2_ + t_β_) S/δ]^2^ [21]**.** δ was the admissible error, and 0.3S was established; S was the population standard deviation. The final calculated sample size of per group was approximately 117. Considering the age range (3–16 years of age), place of residence (rural areas and urban areas) of children and the non-response rate of 20%, the population recruited in the present study was suggested to be more than 4095.

The sample size in the present study (*N* = 4187) was slightly larger than the calculated result (*N* = 4095), and it was enough to observe the group differences between LBC and NLBC.

### Recruitment procedures

A two-step process was conducted to identify the communities. First, 50 communities (22 urban communities and 28 rural communities) were selected from 21 prefecture-level cities. In each prefecture-level city, at least two communities were selected (one in rural areas and the other in urban areas) by using a simple randomization method and random number table. Finally, 10 LBC and 18 NLBC in each age group were randomly selected from each community.

According to the article 16 of the General Principles of the Civil Law of the People’s Republic of China [22] and the article 14 of International Ethical Guidelines for Biomedical Research Involving Human Subjects [23], for participants aged 10–16 years, written informed consents were obtained from both children themselves and one of their current guardians, and for participants aged 3–9 years, written informed consents were obtained from one of their current guardians before the survey. Usually, parents are statutory guardians of minors. However, when both parents were apart from children, the written informed consent form was obtained from one of the current guardians designated by a parent. Participants were guaranteed that their responses in the questionnaire were anonymous and confidential. This study was approved by the Biomedical Research Ethics Review Committee of the School of Public Health, Sun Yat-sen University (Guangzhou, China). Besides, all data were collected through home visit by field investigators who had years of experiences of field investigation and were employed in the field investigation team of the city women’s federation. These investigators were chosen from each city, and were trained uniformly by Guangdong Women’s Federation and project teams in two weeks before the study started.

### Measures

Children’s mental health was measured by either self-reported or parent-reported version of Strengths and Difficulties Questionnaire (SDQ) [24], which aims to assess the behaviour, emotion and relationship in children aged 3–17 years [25]. SDQ consists of 25 items, which were divided into five subscales including emotional symptoms, conduct problems, hyperactivity-inattention, peer problems and prosocial behaviours, with five items in each subscale. Each item scores from zero to two. The aggregate score of all subscales except for prosocial behaviours drive the score of a total difficulties score (TDS) ranging from 0 to 40. Generally, a high score indicates greater difficulties, except prosocial behaviours. It has been proved that the SDQ has good reliability and validity in different cultures [25], including Chinese [26]. In the present study, parent-reported version SDQ was used in 3–9 year-old LBC, and the self-reported version in 10–16 year-old LBC.

### Statistical analysis

The data were entered through Epidata 3.1 software and analysed with SPSS 22.0 statistical software. Chi-square test and the unpaired Student t-test were used to describe the difference in demographics between LBC and NLBC. Secondly, analysis of covariance (ANCOVA) were adopted to evaluate the difference of SDQ scores between LBC and NLBC. Besides, in order to better understand the difference between LBC and NLBC within each age group, participants were divided into three age groups (3–6, 7–9 and 10–16 years) for analysis. That is because children in China start primary school at the age of seven, and children begin puberty around the age of 10 [27], which may result in significant differences within the three age subgroups [28]. Furthermore, we stratified the participants into two groups based on place of residence (rural and urban areas), then analysed the difference between LBC and NLBC within the same areas separately. Finally, stepwise multiple linear regression analysis was used to explore the association between the demographics or the characteristics of being left-behind and the LBC’s emotional, behavioural and relationships problems.

## Results

### Demographic characteristics of LBC and NLBC

Table 1 presents the basic demographic characteristic of the participants. Nearly one-third of participants were LBC, in which 86.7% of them lived in rural areas, and 13.3% lived in urban areas (*p* < 0.001). The percentage of only child in LBC and NLBC were 21.0 and 46.9% (*p* < 0.001). The percentages of paternal or maternal educational level at middle school or below in LBC and NLBC were over 70 and 45% (both *p* < 0.001). LBC’s family average monthly income were lower than that of NLBC (*p* < 0.001). Additionally, Additional file 1: Table S1 presented the basic characteristics of the experience of being left-behind of LBC including age at separation, duration of parent absence, communication frequency, et al. And 26.9% of LBC had a previous experience of being left-behind and were living with both parents during the investigation, while 73.1% of LBC were currently being left-behind. Over 77.8% of LBC were separated from their migrant parents under the age of six. And 52.1% of them were taken care of by grandparents, 35.7% by mother when they were being left-behind.

### Mental health problems in LBC and NLBC according to age groups

The comparison of mental health problems (assessed by SDQ subscales score) between LBC and NLBC was presented in Table 2. Overall, LBC tended to have more difficulties than NLBC, which can be inferred by the higher score of TDS, emotional symptoms, hyperactivity-inattention, peer problems and lower score of prosocial behaviours in LBC (all *p* < 0.05). The participants were next stratified into three age groups (3–6, 7–9 and 10–16 years) to explore the differences within age groups separately. We found that compared with NLBC, LBC scored significantly higher on TDS, peer problems and lower on prosocial behaviours (all *p* < 0.001) in 3–6 years; and higher on hyperactivity-inattention in 7–9 and 10–16 years (both *p* < 0.05). However, no such difference was found after adjusting place of residence, sex, age, only child, average monthly income and both parents’ educational level.

**Table 2:** Comparison of child mental health (SDQ outcomes) between LBC and NLBC by age groups ^a^

Scores of SDQ subscales	LBC	NLBC	*P*^1^	*P*^2^
3–16 years	*n* = 1444	*n* = 2683		
Total difficulties score	12.87 (6.01)	12.26 (6.05)	0.011	0.907
Emotional symptoms	2.79 (2.14)	2.55 (2.19)	0.001	0.482
Conduct problems	2.47 (1.99)	2.45 (1.92)	0.862	0.257
Hyperactivity-inattention	4.12 (2.11)	3.96 (1.99)	0.015	0.620
Peer problems	3.50 (1.65)	3.31 (1.65)	< 0.001	0.809
Prosocial behaviours	5.99 (2.29)	6.17 (2.11)	0.002	0.443
3–6 years	*n* = 568	*n* = 1050		
Total difficulties score	13.66 (5.91)	12.64 (5.70)	< 0.001	0.924
emotional symptoms	3.01 (2.12)	2.65 (2.10)	0.001	0.827
conduct problems	2.50 (1.96)	2.44 (1.82)	0.548	0.181
hyperactivity-inattention	4.58 (2.08)	4.37 (1.90)	0.052	0.834
peer problems	3.58 (1.69)	3.17 (1.64)	< 0.001	0.246
prosocial behaviours	5.45 (2.35)	5.85 (1.99)	< 0.001	0.141
7–9 years	*n* = 414	*n* = 707		
Total difficulties score	12.39 (5.84)	11.98 (5.92)	0.256	0.495
Emotional symptoms	2.66 (2.10)	2.42 (2.17)	0.071	0.768
Conduct problems	2.32 (1.99)	2.28 (1.90)	0.773	0.506
Hyperactivity-inattention	4.07 (2.09)	4.09 (1.94)	0.019	0.166
Peer problems	3.34 (1.57)	3.18 (1.64)	0.108	0.716
Prosocial behaviours	6.26 (2.23)	6.17 (2.10)	0.511	0.280
10–16 years	*n* = 462	*n* = 926		
Total difficulties score	12.32 (6.20)	12.06 (6.52)	0.335	0.753
Emotional symptoms	2.61 (2.18)	2.53 (2.32)	0.504	0.790
Conduct problems	2.55 (2.02)	2.60 (2.03)	0.709	0.879
Hyperactivity-inattention	3.61 (2.05)	3.38 (1.97)	0.038	0.115
Peer problems	3.53 (1.66)	3.56 (1.63)	0.773	0.378
Prosocial behaviours	6.42 (2.15)	6.54 (2.19)	0.488	0.474

*Note. LBC* left-behind children *NLBC* non-left-behind children

^a^ Mental health was assessed by *SDQ*, which includes five subscales and have been detailed described in method. Date are the mean (*SD*). *P*^1^ < 0.05 LBC vs. *NLBC* assessed by independent-samples Student’s t-test; *P*^2^ < 0.05 *LBC* vs. *NLBC* assessed by one-way *ANOVA*, adjusted place of residence, sex, age, only child, average monthly income, father’s educational level and mother’s educational level

### Multiple comparison of mental health problems between LBC and NLBC in rural and urban areas

Considering the possible effects of place of residence on the mental health problems, we further stratified the children into rural and urban group, then analysed the difference between LBC and NLBC after adjustment for sex, age, only child, average monthly income, and both parents’ educational level (Table 3). We found that in rural areas, compared with NLBC, LBC tended to have significantly lower score on prosocial behaviours in whole ages except for 7–9 years (3–16 years, *p* = 0.007, 3–6 years, *p* = 0.030; 10–16 years, *p* = 0.025), higher score on hyperactivity-inattention in 10–16 years (*p* = 0.030), and lower score on conduct problems in 7–9 years (*p* = 0.050). Additionally, in urban areas, LBC had significantly higher emotional symptoms score in 7–9 years group (*p* = 0.029) and higher prosocial behaviour score among the whole samples except for 3–6 years (3–16 years, *p* < 0.001, 7–9 years, *p* = 0.002; 10–16 years, *p* = 0.001).

**Table 3:** Multiple comparison of child mental health (SDQ outcomes) between LBC and NLBC in rural and in urban areas ^a^

	Rural		*P*^1^	Urban		*P*^2^	*P*^3^	*P*^4^
	LBC	NLBC		LBC	NLBC			
3–16 years	*n* = 1276	*n* = 1503		*n* = 195	*n* = 1213			
Total difficulties score	12.97 (5.91)	12.75 (6.05)	0.690	12.17 (6.59)	11.66 (6.01)	0.852	0.003	0.003
Emotional symptoms	2.80 (2.11)	2.72 (2.18)	0.613	2.67 (2.37)	2.32 (2.19)	0.110	0.853	0.006
Conduct problems	2.46 (1.95)	2.53 (1.93)	0.112	2.52 (2.19)	2.36 (1.90)	0.962	0.405	0.616
Hyperactivity-inattention	4.17 (2.11)	4.05 (2.06)	0.542	3.76 (2.08)	3.83 (1.88)	0.387	0.155	0.130
Peer problems	3.54 (1.63)	3.44(1.66)	0.731	3.22 (1.71)	3.14 (1.61)	0.583	0.104	0.039
Prosocial behaviours	5.86 (2.28)	6.20 (2.16)	0.007	6.85 (2.19)	6.15 (2.04)	< 0.001	0.251	0.026
3–6 years								
Total difficulties score	13.90 (5.82)	13.57 (5.55)	0.788	11.77 (6.32)	11.56 (5.68)	0.697	0.101	< 0.001
Emotional symptoms	3.09 (2.08)	2.94 (2.09)	0.559	2.38 (2.37)	2.31 (2.05)	0.683	0.081	< 0.001
Conduct problems	2.53 (1.94)	2.66 (1.80)	0.213	2.31 (2.13)	2.18 (1.81)	0.945	0.396	0.004
Hyperactivity-inattention	4.64 (2.11)	4.60 (1.89)	0.872	4.05 (1.75)	4.10 (1.89)	0.546	0.150	0.002
Peer problems	3.65 (1.69)	3.36 (1.70)	0.170	3.03 (1.60)	2.96 (1.55)	0.804	0.405	0.013
Prosocial behaviours	5.33 (2.37)	5.72 (2.06)	0.030	6.36 (1.96)	6.00 (1.90)	0.116	0.091	0.657
7–9 years								
Total difficulties score	12.35 (5.73)	12.79 (5.73)	0.061	12.63 (6.57)	11.05 (6.00)	0.105	0.220	0.027
Emotional symptoms	2.65 (2.09)	2.66 (2.10)	0.267	2.77 (2.18)	2.15 (2.21)	0.029	0.163	0.081
Conduct problems	2.26 (1.92)	2.40 (1.91)	0.050	2.67 (2.38)	2.15 (1.90)	0.065	0.130	0.843
Hyperactivity-inattention	4.08 (2.07)	4.33 (2.02)	0.111	4.02 (2.26)	3.82 (1.80)	0.898	0.649	0.005
Peer problems	3.36 (1.55)	3.40 (1.67)	0.410	3.18 (1.72)	2.92 (1.58)	0.506	0.862	0.042
Prosocial behaviours	6.08 (2.19)	6.12 (2.11)	0.698	7.33 (2.17)	6.23 (2.10)	0.002	0.003	0.418
10–16 years								
Total difficulties score	12.40 (6.08)	11.93 (6.66)	0.534	11.82 (6.87)	12.26 (6.23)	0.476	0.378	0.181
Emotional symptoms	2.59 (2.12)	2.57 (2.31)	0.727	2.75 (2.49)	2.47 (2.33)	0.257	0.262	0.933
Conduct problems	2.57 (2.01)	2.49 (2.08)	0.603	2.47 (2.05)	2.76 (1.95)	0.120	0.168	0.042
Hyperactivity-inattention	3.67 (2.02)	3.30 (2.04)	0.030	3.29 (2.19)	3.49 (1.87)	0.213	0.105	0.048
Peer problems	3.57 (1.63)	3.58 (1.61)	0.642	3.31 (1.78)	3.54 (1.66)	0.297	0.285	0.781
Prosocial behaviours	6.33 (2.11)	6.74 (2.19)	0.025	6.91 (2.36)	6.24 (2.14)	0.001	0.107	< 0.001

*Note. LBC* left-behind children *NLBC* non-left-behind children

a Mental health was assessed by *SDQ*, which includes five subscales and have been detailed described in method.. Date are the mean (*SD*). *P*1 < 0.05 *LBC* vs. *NLBC* in rural areas, *P*2 < 0.05 *LBC* vs. *NLBC* in urban areas, *P*3 < 0.05 rural *LBC* vs. urban *LBC*, *P*4 < 0.05 rural *NLBC* vs. urban *NLBC* all assessed by one-way *ANOVA*, adjusted sex, age, only child, average monthly income, father’s educational level and mother’s educational level

Furthermore, when we compared the difference of SDQ scores between rural LBC and urban LBC after the same adjustment (Table 3). Results showed that rural LBC had higher TDS score in 3–16 years (*p* = 0.003) and lower prosocial behaviours score at 7–9 years (*p* = 0.003).

### Influence factors of mental health problems in rural and urban LBC

Influence factors of mental health problems in rural and urban LBC were analysed by multiple regression (Table 4). For rural LBC, the score of TDS were significantly negatively associated with age, paternal educational level, duration of parental absence and communication frequency; while positively associated with mother’s educational level and duration of talk per time. And the prosocial behaviours scores were positively associated with siblings, paternal educational level, communication frequency and older age, while negatively associated with duration of parent absence. As for urban LBC, the score of difficulties (including emotional problems, conduct problems and TDS) were found negatively associated with paternal educational level and duration of talk per time. Besides, the score of prosocial behaviours were positively associated with paternal educational level, duration of parental absence and duration of talk per time.

**Table 4:** Regression coefficients for SDQ outcomes on rural and urban LBC ^a^

	Total difficulties score					Prosocial behaviours				Emotional symptoms				conduct problems				hyperactivity-inattention				Peer problems		
	rural			urban		rural		urban		rural		urban		rural		urban		rural		urban		rural		urban
Age group (ref: 10–16 years)																								
3–6 years	0.156***					−0.262***		−0.211		0.137***								0.250***						
7–9 years	0.012					−0.085*		0.253*		0.022								0.108**						
Sex (ref: boys)																		−0.074*						
Only child (ref: yes)						0.102**																		
Monthly income (ref: < 2000 yuan)																								
2000–4999 yuan										0.109**														−0.265
5000 7999 yuan										0.070														− 0.192
8000–12,000 yuan										0.012														−0.439**
> 12,000 yuan										0.046														−0.294**
Father’s educational level (ref: primary school or below)																								
middle school	−0.095			−0.451		0.103*		0.537*		−0.127**		−0.481				−0.496		−0.116*				−0.125*		
high school	−0.140**			−0.592*		0.087		0.516*		−0.088		−0.549*				−0.605*		−0.143**				−0.107*		
college or bachelor	−0.018			−0.488		−0.027		0.674*		−0.065		−0.524				−0.520		−0.031				−0.010		
Mother’s educational level (ref: primary school or below)																								
middle school	0.085													0.134**				0.079						−1.244***
high school	0.154**													0.163***				0.135**						−1.121***
college or bachelor	−0.023													0.000				−0.019						−0.940**
Age at separation (ref: 0–3 years)																								
3–6 years								−0.188																
6–10 years								−0.283*																
older than 10 years								0.300*																
Duration of parent absence (ref: half a year)																								
less than 1 year	−0.017					−0.111*		0.045		−0.016				0.029		0.239		−0.069		−0.061		0.042		0.041
1–2 years	−0.003					−0.112*		0.006		0.051				0.019		0.104		−0.057		−0.121		−0.14		0.232
2–3 years	−0.136**					−0.009		0.015		−0.091				−0.141**		−0.011		−0.151***		−0.109		−0.083		0.281*
more than 3 years	−0.237***					−0.063		0.283*		−0.205***				−0.212***		−0.050		−0.189***		−0.335**		−0.131*		−0.137
Communication frequency (ref: everyday)																								
1–2 times a week	0.129**					−0.074				0.060				0.113**		−0.117		0.163***				0.040		0.130
1–2 times a month	0.077					−0.155***				0.008				0.075		0.070		0.081				0.070		0.267*
1–2 times half a year	0.085*					−0.171***				0.049				0.076*		−0.300**		0.054				0.088*		0.092
1–2 times a year	0.208***					−0.237***				0.124***				0.202***		0.147		0.111				0.171***		0.110
Duration of talk per time (ref: less than 10 min)																								
10–20 min	−0.039		−0.635***				0.090			0.018				−0.128**		−0.729***				−0.712***		0.002		
20–40 min	0.087		−0.385**				0.096			0.110*				−0.028		−0.541***				−0.453**		0.108*		
one hour	0.090*		−0.189				0.003			0.101*				0.050		−0.426**				0.343**		0.122**		
more than one hour	0.154***		−0.295*				0.295*			0.188***				0.117**		−0.370**				−0.276*		0.125**		
Visit frequency (ref: every weekend)																								
every month																		0.075						
1–2 times half a year																		0.004						
1–2 times a year																		−0.010						
less than once a year																		0.096*						
Migrant worker (ref: both)																								
Father absent	0.116***									0.089*				0.078*				0.132***						
Mother absent	0.038									0.028				0.077*				0.058						

*Note.* Data are standardized regression coefficients. ^a^ Data are Beta assessed by stepwise multiple linear regression analysis

* *p* < 0.05 ** *p* < 0.01 ***p < 0.001

## Discussion

The present study investigated the status of mental health and its influencing factors in both rural and urban LBC aged 3–16 years. The results showed no statistically significant difference in the difficulties of mental health problems between LBC and NLBC on the whole participants as well as in rural areas and in urban areas within the same age after adjustments were made. However, urban LBC tended to have the most prosocial behaviours, while rural LBC had the least prosocial behaviours not only in the whole age group but also in different age subgroups. Furthermore, we also found that higher paternal educational level and longer duration of parental absence were associated with less mental health problems in both rural and urban LBC. Besides, two migrant parents, shorter duration of talk per time but higher communication frequency were found associated with less mental health problems in rural LBC.

Contradicted with most of the existing studies [7, 15], the present study displayed no significant differences in mental health problems and prosocial behaviours between NLBC and LBC in the whole age and in the age subgroups, respectively, after controlling for the major confounding variables. The population- and demographic-limitation of the prior studies might contribute to the inconsistence. Prior studies were mostly conducted at school communities of rural areas, failing to control the confounders [17, 18] such as family backgrounds (family income, parental educational level, only-child, etc.), living environments, and social relationships, while the present study tried to make them controlled as far as possible in analysis. Besides, Shalhevet etc. found that great grandparent involvement was associated with better social skills and less emotional or behaviour problems of adolescent, especially in the lone-parent and step-families [29, 30]. Meanwhile, according to the attachment theory [30–32], that the construction of secure attachment relationship with alternate caregivers can be the foundational support for children’s development of mental health [33]. It was possible that they were well taken care of by alternate caregivers (mostly were grandparents) [29] when they were separating from one or two parents.

Meanwhile, when it comes to the subscales of the mental health problems, interesting result was observed. Compared with NLBC in the same place, LBC were not always the vulnerable groups, they also had some strengths: rural LBC tended to have less conduct problems in 7–9 years and urban LBC had more prosocial behaviours in 7–16 years, after adjusting for parental educational level and factors of family environment. The possible explanation might be that the development of human being is a lifelong process of change in the abilities to adapt to the situations one selected [32], and many elements like living environments, the person/cognition and education during childhood [34] are related to maturation. Thus, we speculate that the effects of separation from migrant parents might be mitigated by other factors in children’s life, which still needs further investigation.

Furthermore, the present study also showed that paternal educational level was negatively correlated with the problems of LBC’s mental health, which was in line with the previous studies that paternal education level was unique for children’s language and cognitive development [35, 36]. In Chinese culture, it is believed that fathers are responsible for economic support and discipline maintenance, while mothers for nurturing. And this concept is far more popular in rural China [37]. However, urbanization and maternal employment are changing these attitudes. Fathers, especially those college-educated, are better understand the importance of intimate relation between father and children, and are more involved in their children’s life [36, 38]. Fathers’ frequent and positive involvement with children can promote the well-being of children’s cognitive and social development [39]. However, in rural areas, women with high education level tend to be eager to pursue for self-value than those with lower education level [40]. And this may result in less interaction between mother and child which was proved to have negative effect on children’s wellbeing [41, 42]. Considering the lack of relevant data in the present study, we can’t tell the exact mechanism. More researches are needed in the future studies.

Besides, we also found that rural LBC with longer duration of parental absence and with both migrant parents tend to have less emotional problems, hyperactivity-inattention, conduct problems and peer problems. To LBC, longer duration of parent absence and both migrant parents means they spent more time with the alternate caregiver. Raikes [43] found that the more time infants spent with the same caregiver, the more secure attachment relationship with caregiver will be formed. Additionally, Howes, etc. [44] found that children with more caregivers at the age of one to four were more likely to be aggressive with peers than those with fewer caregivers. Every time the migrant parent came home, LBC needed to construct a new child-parent relationship instead of continuing the previous one [31], which might become new challenges to LBC [45].

Furthermore, the present study also found that more frequency but a short talk with migrant parents had close relationship with rural LBC’s less emotional symptoms, less conduct and peer problems. This result partly supports the findings presented by Yu Guang etc. [46] that communication with migrant parents for over 5 min with suitable topics could significantly decrease the depressive symptoms in rural LBC. However, the association with mental health outcomes might be different in urban LBC subgroups. Considering the small sample size of urban LBC, some variables may not able to reach the statistical significance [21]. Considering that and the complex mechanism of these correlations, more research on these topics are needed. Besides, parenting intervention program should be developed for LBC in various socio-demographic groups.

There are some limitations in the present study. First, as a cross-sectional survey, the results can only show correlations instead of causations. Longitudinal design should be applied in future study to explore and analyse the causal relationship between mental health and other influence factors. Second, the present study did not focus much on the mechanism of how the details of being left-behind interfere with mental health of LBC. Thus, further studies are needed to find out the essential characteristics which influence it in left-behind children. Third, all of these subjects of the project were drawing from one province of south China. Therefore, it should be more cautious when extrapolating the results to the whole country.

## Conclusions

The present study demonstrated that in general, no differences of mental health problems were found between LBC and NLBC. According to our results, those rural LBC with both migrant parents, longer duration of parental absence, shorter duration of talk per time but more communication frequency, and higher paternal educational level tend to have better development of mental health. Thus, we suggest that more attention should be paid on improving the stability of caregivers and the effectiveness of parent-child communication of Chinese rural LBC when making policies in the future.

## Supplementary information

**Additional file 1: Table S1.** Basic characteristics of the experience of being left-behind. Table S1 presents the basic characteristics of the experience of being left-behind of LBC and the constitution of each factor. (DOCX 19 kb)

## Data Availability

The datasets used and/or analyzed during the current study are available from the corresponding author on reasonable request.

## Abbreviations

ANCOVA: Analysis of covariance
LBC: Left-behind children
NLBC: Non-left-behind children
SDQ: Strength and Difficulties Questionnaire
TDS: Total difficulties score
