# Supplementary material for: Mental health and its influencing factors among left-behind children in South China: a cross-sectional study
Source: BMC Public Health. 2019 Dec 23;19:1725. doi: 10.1186/s12889-019-8066-5 (PMC6929312; doi:10.1186/s12889-019-8066-5)
Supplement: Supplementary file 1 — Additional file 1: Table S1. Basic characteristics of the experience of being left-behind. Table S1 presents the basic characteristics of the experience of being left-behind of LBC and the constitution of each factor. (DOCX 19 kb) [file 12889_2019_8066_MOESM1_ESM.docx]

Table S1: Basic characteristics of the experience of being left-behind ^a^

| **Variables** | **LBC (**n=1471) | **Rural (n=1276)** | **Urban (n=195)** |
| --- | --- | --- | --- |
| The period of being left-behind |  |  |  |
| previous | 393(26.9%) | 319(25.1%) | 74(38.5%) |
| current | 1069(73.1%) | 951(74.9%) | 118(61.5%) |
| Age at separation |  |  |  |
| 0-3 years | 524(40.3%) | 461(40.5%) | 63(39.4%) |
| 3-6 years | 487(37.5%) | 433(38.0%) | 54(33.8%) |
| 6-10 years | 213(16.4%) | 186(16.3%) | 27(16.9%) |
| older than 10 years | 75(5.8%) | 59(5.2%) | 16(10.0%) |
| During of separation |  |  |  |
| half a year | 195(14.7%) | 307(26.4%) | 33(19.9%) |
| less than 1 year | 340(23.1%) | 206(17.7%) | 32(19.3%) |
| 1-2 years | 238(17.9%) | 182(15.6%) | 20(12.0%) |
| 2-3 years | 202(15.2%) | 311(26.750 | 43(25.9%) |
| more than 3 years | 354(26.6%) | 307(26.4%) | 33(19.9%) |
| Migration type |  |  |  |
| father-only | 692(47.4%) | 583(46.0%) | 109(56.5%) |
| mother-only | 144(9.9%) | 119(9.4%) | 25(13.0%) |
| both-parent | 624(42.7%) | 565(44.6%) | 59(30.6%) |
| Main caregiver when left-behind |  |  |  |
| grandparents | 759(52.1%) | 678(53.6%) | 81(42.0%) |
| father | 102(7.0%) | 85(6.7%) | 17(8.8%) |
| mother | 521(35.7%) | 438(34.6%) | 83(43.0%) |
| brother/sister | 20(1.4%) | 17(1.3%) | 3(1.6%) |
| relatives | 18(1.2%) | 11(0.9%) | 7(3.6%) |
| bording | 38(2.6%) | 36(2.8%) | 2(1.0%) |
| Contact tools |  |  |  |
| telephone | 1260(95.2%) | 1114(95.9%) | 146(90.1%) |
| computer/ video | 30(2.3%) | 23(2.0%) | 7(4.3%) |
| letters | 13(1.0%) | 6(0.5%) | 7(4.3%) |
| others | 21(1.6%) | 19(1.6%) | 2(1.2%) |
| During of contact per time |  |  |  |
| less than 10 min | 232(17.7%) | 209(18.1%) | 23(14.5%) |
| 10-20 min | 540(41.2%) | 467(40.5%) | 73(45.9%) |
| 20-40 min | 345(26.3%) | 309(26.8%) | 36(22.6%) |
| one hour | 104(7.9%) | 92(8.0%) | 12(7.5%) |
| more than one hour | 91(6.9%) | 76(6.6%) | 15(9.4%) |
| Visit frequency |  |  |  |
| every weekend | 217(16.5%) | 172(14.9%) | 45(28.5%) |
| every month | 287(21.8%) | 240(20.7%) | 47(29.7%) |
| 1-2 times half a year | 537(40.8%) | 500(43.2%) | 37(23.4%) |
| 1-2 times a year | 237(18.0%) | 212(18.3%) | 25(15.8%) |
| less than once a year | 38(2.9%) | 34(2.9%) | 4(2.5%) |

*Note.* LBC= left-behind children.

^a^ Data are n (%).
